# Wernicke’s Encephalopathy in a Patient Without Chronic Alcohol Abuse

**DOI:** 10.5811/cpcem.2016.12.32769

**Published:** 2017-03-14

**Authors:** Darshan Thota, Sherri Rudinsky

**Affiliations:** Naval Medical Center, Department of Emergency Medicine, San Diego, California

## Abstract

Wernicke’s encephalopathy (WE) is traditionally seen in the emergency department in patients with chronic alcohol abuse. WE can result in significant morbidity and mortality if untreated, making early diagnosis and intervention paramount. We discuss a case of WE in a 63-year-old female with no history of chronic alcohol abuse, who presented with bilateral opthalmoplegia that resolved after intravenous thiamine administration. This case report highlights the varied clinical settings other than chronic alcohol abuse in which the diagnosis of WE should be considered.

## INTRODUCTION

Wernicke’s encephalopathy (WE) is traditionally associated with chronic alcohol abuse and characterized by some combination of ataxia, ophthalmoplegia, and altered mental status. Chronic alcohol abuse is the most commonly recognized presentation in the emergency department (ED). However, WE has been increasingly recognized in other high-risk clinical settings such as anorexia, post gastric bypass surgery, hyperemesis, AIDS, TPN, and malignancy. WE is still greatly under-recognized based on autopsy data.^1^ Atypical presentations are important to recognize because treatment with thiamine can prevent serious adverse effects such as permanent mental impairment and coma. We present a case of one such atypical presentation to highlight the various risk factors and clinical settings in which WE can occur.

## CASE REPORT

A 63-year old-female presented to the ED with three days of headache, diplopia, and gait instability. The symptoms began while the patient was watching television and progressively worsened over three days. The headache was described as a pressure, located on the left temple without associated neck stiffness or photophobia. The diplopia was binocular and horizontal. She described her gait instability as “walking like I am drunk.” She reported decreased oral intake over the previous day due to “not feeling well.” Recent medical history included community-acquired pneumonia, currently on day 5 of 10 of moxifloxacin. She had no recent travel or changes in her daily activities. The patient had no history of stroke, bariatric surgery or recent vomiting. The patient and family denied a history of alcohol use.

On exam, blood pressure 139/80, heart rate 67, respiratory rate 16, room air oxygen saturation 98% and temperature 97.3 degrees Fahrenheit. She was well nourished, well developed and in no acute distress. Her head was atraumatic with moist mucous membranes. The patient was awake, alert, and oriented to person, place and time. Her eyes were anicteric and the left eye displayed ptosis. Her left pupil was sluggish compared to the right, but both were reactive. Uncorrected visual acuity was 20/60 OD and 20/60 OS. Left eye demonstrated complete paralysis of extraocular movements; right eye demonstrated 10–15 degrees of movement in all directions, and nystagmus in right eye on lateral gaze. The remaining cranial nerves were intact. She had intact strength and sensation to light touch throughout the upper and lower extremities. Cerebellar testing was remarkable for past pointing on the left with finger to nose, and her gait was characterized by small, unsteady steps with ambulation in the ED. The remainder of her exam was unremarkable.

Ancillary testing in the ED was remarkable for a white blood cell count of 14,400/uL, hemoglobin of 15.0 g/uL, hematocrit of 43.4%, and platelet count of 527,000/uL Chemistry panel, liver function studies and erythrocyte sedimentation rate were all within normal limits.

An emergent, non-contrast head computed tomography (CT) was performed, which did not show an acute intracranial hemorrhage, mass effect or midline shift. A brain and neck magnetic resonance imaging and angiography (MRI/MRA) were obtained, which did not demonstrate a cortical lesion, aneurysm or other vascular defect to explain her symptoms. Her case was discussed further with neurology and she was started on high-dose thiamine for concerns of WE and admitted to the internal medicine service for further evaluation. No thiamine level was obtained during her initial admission.

Her symptoms did improve after parenteral high-dose thiamine. She was discharged on hospital day three with continued oral thiamine supplementation. At her one-month neurology follow-up appointment she still had a mild ophthalmoplegia but was otherwise back to her baseline functional status.

## DISCUSSION

The symptom cluster of ataxia, opthalmolplegia, and encephalopathy was first described as “polioencephalitis hemorrhagica superioris” by Carl Wernicke in 1881 in a 20-year-old female who developed pyloric stenosis and projectile vomiting after an unintentional sulfuric acid ingestion.^2^ Thiamine deficiency was not identified as the underlying cause of WE until the 1940s.^2^ In the United States, WE is most commonly recognized in the setting of nutritional deficiencies related to chronic alcohol abuse. The prevalence of WE is 2% in the general population but up to 12.5% in chronic alcoholics.^3^ However, Wernicke’s original case and our patient both highlight the importance of considering this diagnosis in clinical settings other than chronic alcohol abuse.

Any patient with a nutritional deficiency has the potential to develop WE. In one retrospective autopsy study, 12 of 52 (23%) cases of WE did not occur in alcoholics.^4^ A growing number of predisposing factors and clinical settings have been associated with the development of WE and have been reported in the literature to include anorexia nervosa, gastrointestinal surgery including gastric bypass surgery, cyclic vomiting, hyperemesis gravidarum, cancer and chemotherapeutic agents specifically 5-Fluorouracil and Cisplatin, peritoneal and hemodialysis, chronic TPN, thyrotoxicosis and AIDS.^5^–^13^ Given the varied clinical presentations, a high index of clinical suspicion is needed.

WE is a clinical diagnosis characterized by ophthalmoplegia, mental status changes, and unsteadiness of stance and gait. This symptom triad is only seen in up to 16% of cases.^14^ No specific lab tests or radiographs are pathognomonic in diagnosing WE. Only one or two symptoms from the triad may be present upon initial presentation and symptoms vary for each component. Nystagmus is the ocular abnormality most commonly recognized and occurs in up to 29% of patients; it can be either horizontal or vertical in direction.^1^ Other reported ocular abnormalities include ophthalmoplegia, gaze palsies, anisocoria, and retinal hemorrhages.^1^

Changes in mental status are common at presentation, occurring in 80–90% based on retrospective reviews.^1^ These changes can range from confusion, memory difficulty, mild delirium, confabulation, apathy, decreased concentration, and coma. Neuropsychiatric symptoms, such as hallucinations, delusions, and agitation, which may mimic acute psychosis, have also been described.^1^ Ataxia can present as both truncal and gait based with loss of vestibular equilibrium affecting the cerebellar vermis in 23% of patients.^1^

Given the varied spectrum of disease, WE can be a difficult diagnosis to make. A combination of four operational criteria has demonstrated improvement in the identification of WE in chronic alcoholics.^15^ Demonstrating two of four criteria – dietary deficiencies, oculomotor abnormalities, cerebellar dysfunction, and altered mental status or mild memory impairment – results in a sensitivity and specificity of 100% and 98% respectively. Expanding the definition to include dietary deficiencies improved the sensitivity in diagnosing WE from 22% to 85% in one retrospective review.^15^ Our patient met two of the four criteria, lacking altered mental status and dietary deficiency. Expanding the traditional definition of WE to include any dietary deficiency and recognizing the spectrum of presenting symptoms will improve provider recognition of WE.

WE remains a clinical diagnosis without definitive laboratory studies, imaging, cerebrospinal fluid or electroencephalograph findings. Though not overly sensitive, MRI can sometimes help in confirming the diagnosis of suspected WE. The most specific MRI findings are cytotoxic and vasogenic edema in the third ventricle, mammillary bodies, midbrain tectal plate, and periaqueductal area.^16^ These changes typically occur within 2–3 weeks of thiamine deficiency.^1^ The sensitivity and specificity of MRI for diagnosing WE have been reported as 53% and 93% respectively.^17^ The MRI findings noted above are not specific to WE and may be seen in other disease processes.^18^

WE is a medical emergency and left untreated can progress to Korsakoff syndrome and beriberi. Korsakoff is seen in 80% of acute Wernicke’s episodes and is characterized by confabulation and deficits in working memory.^19^ Beriberi can either be cardiogenic resulting in heart failure and edema, or neuropathic resulting in paresthesias. With the significant morbidity of Korsakoff syndrome and reported 17% mortality rate of WE, early treatment with thiamine is of key importance. Classic teaching dictates correction of hypoglycemia prior to thiamine administration to prevent worsening WE. However, a recent review of 14 articles found no data to support this recommendation and stressed importance of treating symptomatic hypoglycemia acutely.^20^ There is a paucity of data regarding the optimal intravenous or enteral thiamine replacement strategies. However, 500 mg thiamine dissolved in 100 ml of normal saline infused intravascularly over 30 minutes acutely in the ED followed by a maintenance dose of 500 mg IV three times a day for 2–3 days is thought to be optimal treatment for WE. Clinical improvement is anticipated in 2–3 days and if seen, 250 mg orally daily is then given for 3–5 days.^1^ Nystagmus and ophthalmoplegia typically resolve within hours to days, while ataxia and mental status changes can take up to weeks to months to resolve completely.^5^

Several other disease states may mimic WE. In our differential diagnosis we considered intracranial mass lesions, hemorrhage, infarcts, meningitis and other infection to include syphilis and Lyme disease, glaucoma, hypoglycemia, electrolyte abnormalities, Miller Fisher variant of Guillain-Barré syndrome, Horner’s syndrome, and multiple sclerosis. Our patient’s CT showed no structural disease. Her physical exam and history were inconsistent for meningitis and she had no recent travel to suggest Lyme. In our patient, an extensive evaluation for other etiologies to explain her symptoms was negative. Though moxifloxacin in known to cause peripheral neuropathies, it is unlikely to be the cause of the patient’s opthalmoplegia. Thyroid disease and HIV can cause ocular symptoms and cranial neuropathies; however, both HIV and thyroid function tests were negative in our patient.

## CONCLUSION

Our case report highlights the varied clinical settings in which WE may present and the need to entertain the diagnosis and look for predisposing factors in any patient presenting with ataxia, ophthalmoplegia or mental status changes regardless of history of chronic alcohol use. Given the high morbidity and mortality and simple therapeutic interventions, emergency physicians have the ability to make a timely and dramatic impact in the outcome of these patients’ disease course.
